# Decrease of Quality of Life, Functional Assessment and Associated Psychological Distress in Patients with Hypoallergenic Total Knee Arthroplasty

**DOI:** 10.3390/jcm9103270

**Published:** 2020-10-12

**Authors:** Pilar Peña, Miguel A. Ortega, Julia Buján, Basilio De la Torre

**Affiliations:** 1 Orthopedic Surgery and Traumatology Service, Virgen de la Luz Hospital, 16002 Cuenca, Spain; pilarpf1204@yahoo.es; 2 Departments of Medicine and Medical Specialities, Faculty of Medicine and Health Sciences, University of Alcalá, Alcalá de Henares, 28801 Madrid, Spain; mjulia.bujan@uah.es; 3 Ramón y Cajal Institute of Sanitary Research (IRYCIS), 28034 Madrid, Spain; bjtorre@gmail.com; 4 Department of Surgery, Medical and Social Sciences, Faculty of Medicine and Health Sciences, University of Alcalá, Alcalá de Henares, 28801 Madrid, Spain; 5 Service of Traumatology, University Hospital Ramón y Cajal, 28034 Madrid, Spain

**Keywords:** total knee arthroplasty, metal allergy, oxinium, quality of life, functional capacity, SF-12, WOMAC, Euro-Qol-5D L-VAS, psychological distress

## Abstract

Total knee arthroplasty (TKA) is the final treatment for knee osteoarthritis, and 15–30% of patients show little or no improvement. This high percentage is related to aspects of the surgical technique, the selected implant, and specific patient characteristics. The aim of this study was to analyze whether there are differences in quality of life (QoL) and functional capacity among patients undergoing TKA with conventional implants compared to those treated with hypoallergenic oxinium implants. A pragmatic clinical study was carried out that included patients who underwent TKA between January 2013 and December 2015. During this period, 245 knees in 228 patients were treated. Eleven patients were excluded, leaving a sample of 161 conventionally treated knees, 72 knees treated with hypoallergenic implants, and one patient who received both implant types. In all patients, QoL and functional capacity were measured with the WOMAC index, the SF-12 questionnaire, and the Euro-Qol-5D L-VAS. We also assessed the psychological distress of each patient and related the findings to the functional results. The differences in QoL were tested using ANCOVA and propensity score matching (PSM) models adjusted for sex, age, weight, psychiatric history and associated complications. Patients who underwent TKA using conventional prostheses had significantly better scores on the total WOMAC index and in the pain domain (*p* < 0.05) than those who received hypoallergenic prostheses, but no significant differences were observed for the other domains in the ANCOVA. In contrast, with the PSM, we also found statistically significant differences in the difficulty domain of the WOMAC. Significant differences were found for the SF-12 mental health questionnaire results (*p* = 0.038), but the same did not occur for the physical health domain in the ANCOVA and PSM. We also found statistically significant differences in the Euro-Qol-5D index results (*p* = 0.041), but not in the VAS scale scores for the same questionnaire in the ANCOVA, and we did not find significant differences in either with the PSM. Patients with metal allergies and those who present psychological distress had WOMAC, SF-12, and Euro-Qol-5D results that were statistically significantly worse than those of patients who received conventional implants. Patients who underwent hypoallergic TKA had lower scores on the QoL and functional capacity scales than patients who received conventional Cr–Co implants. Additionally, patients with psychological distress had worse results on the questionnaires, and those with a metal allergy had even lower scores; the differences were statistically significant.

## 1. Introduction

Osteoarthrosis of the knee is a progressive and complex degenerative joint disease that produces chronic pain, reduces mobility, and influences quality of life and work capacity, as well as psychosocial interaction [1,2]. Total knee arthroplasty (TKA) is the final treatment for knee osteoarthritis, reducing pain and improving the quality of life of patients [3,4,5,6,7], but between 15% and 30% of patients who undergo TKA have little or no improvement [6,7,8]. This high percentage involves factors related to the surgical technique [5,8], patient characteristics [1,2,9], and the selected implant [2,10]. On the one hand, we know that specific local and systemic adverse effects of the implant due to corrosion and wear are cause for concern [9]; among these, allergies to metals, mainly nickel, are the most controversial. The prevalence of allergies to nickel is approximately 20% of the general population, and it is higher among women. To a lesser extent, we also found hypersensitivity to other metals such as cobalt (6–9%) and chromium (2–6%) [10]. Similarly, skin hypersensitivity to metals has been reported in 25% of patients with arthroplasties that function well and in up to 60% of those with arthroplasties that function poorly [11,12]. Whether the preoperatively identified skin hypersensitivity predisposes a patient to metal orthopedic implant problems is currently unclear. The gold standard test for the diagnosis of metal hypersensitivity is the skin patch test, with high sensitivity but that can produce false positives. Hallab et al. [13] have observed that skin patch tests are highly subjective, not standardized, limited to skin tissues rather than deep, and perhaps most importantly, an assessment of an exposure mechanism in which Langerhans cells initiate a hypersensitivity reaction, which differs from macrophages or a lymphocyte-mediated response that occurs in the periprosthetic environment. However, despite the large percentage of patients with metal sensitivity (20–30% of the general population), there are few cases of real hypersensitivity to metal implants in the periprosthetic tissue, around 1% of patients with a confirmed metal allergy; nonetheless, the issue is still under debate and should be considered a diagnosis by exclusion [14,15]. Despite the existing controversy, many authors advise implanting an antiallergic prosthesis [10,11]. On the other hand, we know that the outcomes of TKA can be related to psychological aspects of the patient; up to 25% of patients undergoing TKA may have psychological distress [16], a term that encompasses a clinical set of psychological symptoms that include anxiety, depression, and somatization [17]. Given the complexity of these two specific patient-related aspects and the scarcity of studies that compare the outcomes of conventional and antiallergic implants, we aim to study their results using three quality of life (QoL) and functional capacity questionnaires (WOMAC, SF-12, and Euro-QoL-5D L-VAS) and relate them to psychological aspects of the patient. Therefore, the main objective of this study is to analyze whether patients with metal allergies undergoing treatment for knee osteoarthritis with total arthroplasty with antiallergic oxygen prosthesis are more likely to have worse clinical results than patients undergoing conventional arthroplasty with Cr–Co. As a secondary objective, we consider whether suffering from psychological distress influences the results in patients who are allergic and not allergic to metals.

## 2. Patients and Methods

### 2.1. Study Design

A retrospective pragmatic clinical study was conducted to make decisions in the clinic and measure the effectiveness of interventions as they are routinely used. The study was approved by the Clinical Research Ethics Committee (REG: 2017/PI0617). All patients were informed about the purposes of the study, and verbal informed consent was obtained from each of the participants.

### 2.2. Patients

**Figure 1:**
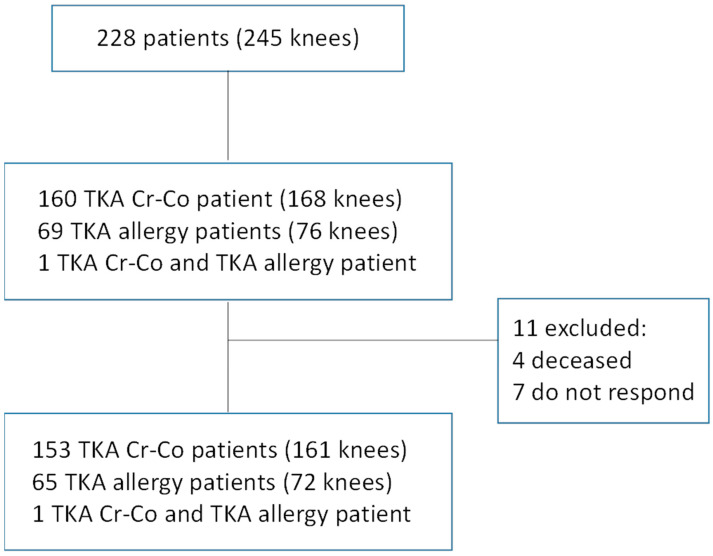
Patient flow chart. Total knee arthroplasty (TKA).

The patients underwent TKA surgery between January 2013 and December 2015. During this period indicated, 245 knees in 228 patients were treated at our hospital: 168 knees were treated with Cr–Co conventional TKA, 76 knees were treated with hypoallergenic oxinium TKA, and one patient received a Cr–Co implant in one knee and an oxinium implant in the other. Eleven patients were excluded (4 who died and 7 who did not respond calls), and the final sample comprised 161 conventional TKAs, 72 hypoallergenic TKAs, and one patient who underwent TKAs in both knees using one of each type of implant (Figure 1). As it was a pragmatic clinical study, where we measured the effectiveness of a treatment, in this case knee osteoarthritis, all patients were included for the study, except for patients under 18 years of age, those who had previously undergone TKA, and those who had not wanted to answer the questionnaires, did not answer the calls, or had died.

The data collected from each patient were age, sex, body mass index (BMI), weight, type of implant (conventional/hypoallergic), laterality (right/left), psychiatric history at the time of the intervention, time since the intervention (months), and associated complications.

The high number of hypoallergenic implants used can be explained: From external consultation programs for knee pathology, many patients were referred to a dedicated clinic outside the general hospital of the public health system. The surgeon who evaluated these patients asked each of them about possible metal allergies. If a patient responded affirmatively, a skin patch test was performed, and patients with positive skin tests were referred back to the general hospital for implantation of a hypoallergenic TKA.

The patients were operated on by the same team of orthopedic surgeons under the same conditions: epidural anesthesia, prophylactic antibiotics (cefazolin 2 g IV or vancomycin 1 g) 30 or 60 min before the skin incision, respectively, under preventive ischemia and using an anterior approach with medial para-patellar arthrotomy. The implanted prostheses were the same except for the material of the femoral implant component, which was either conventional Cr–Co-cemented posterior stabilized (PS) Genesis II or oxinium-cemented PS Genesis II (both from Smith & Nephew, Memphis, TN, USA). The hypoallergenic femoral implant was chosen over the conventional femoral implant component if patients had a positive reaction to the skin patch test performed by the allergy service. The drain was removed 24 h after the operation. All patients began early ambulation with two canes and rehabilitation 24 h after the intervention and after discharge from hospital for a duration of 6 weeks. Outpatient check-ups were performed at 4 weeks, 3, 6, 12, and 24 months after the intervention.

### 2.3. Questionnaires Used

QoL and functional capacity were assessed using three questionnaires: the Western Ontario McMaster Arthritis Index (WOMAC) [2,9,16,18,19,20], the Short Form-12 Health Questionnaire (SF-12) [18,21,22,23,24,25], and the Euro-QoL-5D L-VAS questionnaire [26,27]. To evaluate psychological distress, we worked with the Psychiatry Service to develop a scoring system based on three parameters: psychiatric history, type of pathology, and number of drugs (Table 1). These data were collected retrospectively; thus, we decided to develop a score to avoid the time biases that could have occurred with the use of standardized scales such the Hamilton, Goldberg, or PHQ-15 questionnaires, which collect data prospectively.

The data were collected by one of the authors (MPPF) by reviewing the clinical history of each patient and by a nurse external to the orthopedic surgery service through phone calls. The demographic and psychological distress data were collected retrospectively, while the questionnaires were completed after the study began.

### 2.4. Statistical Analysis

Statistical analyses were performed using SPSS software, version 24 (SPSS, Inc., Chicago, IL, USA). The results are presented as the mean ± standard deviation (SD) or total value and percentage. We performed two statistical analyses, the analysis of covariance (ANCOVA) and the propensity score matching (PSM). To evaluate the differences in QoL parameters between the conventional and hypoallergenic prostheses groups, the ANCOVA and PSM was adjusted for age, sex, weight, previous psychiatric history and associated complications. ANCOVA was performed to compare the WOMAC index, SF-12 scores, and the Euro-Qol-5D index between the groups of patients (TKA with a conventional prosthesis or with a hypoallergenic prosthesis), with adjustment for age, sex, weight, age at intervention, and history of psychiatric and associated complications. Pearson’s chi-squared statistical analysis was also performed to assess the correlation between the final psychological distress score by type of implant and Student’s t test for independent samples to analyze whether psychological distress influenced the questionnaires results for each group. A *p*-value <0.05 represented a statistically significant difference.

## 3. Results

### 3.1. All Patients Characteristics

Table 2 presents the descriptive characteristics of the initial sample of 245 treated knees. A total of 59.2% of the sample in the conventional TKA group were women, compared with 92.1% in the hypoallergenic TKA group (*p* < 0.001). The scores on the WOMAC total and pain scales were significantly higher, and the mental and physical components of the SF-12 and the Euro-Qol-5D index, and the VAS were significantly lower among the patients who received hypoallergenic prostheses (*p* < 0.05).

Researchers’ experience says that both arthroplasties (conventional and hypoallergenic) are the same. We observe that the value of the t-test statistic is 0.438 with a *p*-value of 0.000 (Table 3), so we reject the statement and conclude that the two implants are different.

### 3.2. Patients with Antiallergic Prostheses Show a Change in Their Assessment of Their QoL

To evaluate the differences in QoL parameters between the conventional and hypoallergenic prostheses groups, the ANCOVA was adjusted for age, sex, weight, previous psychiatric history and associated complications (Table 4). The scores for the WOMAC total scale and pain domain, and the mental component of the SF-12 and the Euro-Qol-5D index were significantly lower among the patients treated with hypoallergenic prostheses. In general, the patients in the hypoallergenic TKA group showed worse QoL in the ANCOVA.

After the individuals were paired using PSM (Table 5), it was observed that the scores on the total WOMAC scale and the WOMAC pain and difficulty subscales were higher and SF-Mental, Euro-Qol-5D, and VAS Euro-Qol-5D were lower for those who received hypoallergenic prostheses; that is, this group had worse results than the group treated with a conventional TKA, and the difference was statistically significant (*p* < 0.05).

### 3.3. Psychological Distress with Using Hypoallergenic TKA

Regarding severity, the patients whose final psychological distress score was mild comprised similar proportions of the sample (8.3 and 8.4% of the patients in the study, respectively) (Table 6).

The final psychological distress score according to implant type is shown in Table 7; 18.9% of patients who received a hypoallergenic TKA had severe distress compared with 4.4% of those who underwent conventional TKA. A statistically significant difference in distress was observed between the two groups (*p* = 0.041): in the hypoallergenic implant group, the number of patients with severe pathology was much higher than that in the conventional group (18.9% versus 4.4%) (Table 8).

When examining the results of the conventional implant group, the WOMAC total score, the SF-12 physical and mental subscale scores, the EuroQoL-5D index, and the L-VAS indicated worse results in patients who had previous psychological distress, although the differences were not statistically significant except in the case of the Euro-Qol-5D L-VAS (Table 9). In the antiallergic group, lower parameters were also obtained by patients who suffered from psychological distress, and these differences were statistically significant, in contrast with the findings for the conventional group.

## 4. Discussion

To the best of our knowledge, this is the first study comparing the QoL, functional capacity, and psychological distress of TKA patients treated with a conventional implant and those treated with a hypoallergenic implant where all the patients who self-reported metal hypersensitivity underwent a skin patch test. Patients with a positive patch test were operated with an oxinium implant, while those who tested negative or did not self-report hypersensitivity were operated with conventional TKA. The results show worse functional results for patients who received hypoallergenic implants, worse if the patient also has psychological distress.

The role of metal allergies, especially nickel [10], in implant selection is controversial since it is not clear that a positive preoperative skin patch test for metal hypersensitivity should be interpreted as a true allergy to the metals in orthopedic implants [28]. It is so controversial that Innocenti et al. [15] affirm that there are few cases of real hypersensitivity to metal implants, so this hypersensitivity should be considered as a diagnosis of exclusion, mainly with low-grade infections by low-virulence pathogens, with nonspecific symptoms, false-negative cultures, and inconclusive blood tests [8,15]. Paradoxically, the majority of people with confirmed sensitivity to the metals in the prosthesis before undergoing TKA do not have any adverse events related to the implant. Bravo et al. [29] demonstrated that there was no association between a positive skin patch test and the implantation of a conventional TKA. Patients operated on with conventional TKA and positive patch test did not have a higher failure, reintervention, or revision rate after the implantation of the standard TKA than those patients with negative patch test and the control group. Similarly, in 16 patients with nickel allergies who underwent a TKA with an implant containing nickel, Carlsson et al. [30] showed that after 6 years, none of the patients had complications. Granchi et al. [10] demonstrated that there is good diagnostic precision with the patch test that has a sensitivity value of 1 and a null negative probability ratio, which provides evidence that the skin patch test is very sensitive for metal. What is unclear is whether a positive patch test for metal hypersensitivity represents a “true” metal allergy. In reality, there are false-negative results to metal allergy tests despite it being a very sensitive test. If the latter is true, then we conclude one of the two scenarios: it is possible that cutaneous hypersensitivity to metal is not related to deep allergies or that the patch test is not a good tool for determining deep hypersensitivity. It is very possible that both scenarios are correct and consequently confirm that the patch test may be unnecessary for these allergic patients. Although a positive patch test shows a strong correlation, a causal relationship between dermal reactions and implant failure has not been established as it cannot predict the function of ATR [24].

The study most similar to this work was developed by Nam et al. [31]. As our results show, the values of the physical component of the SF-12 did not show significant differences in postoperative improvement in either group, although the hypoallergenic TKA group had lower scores. The postoperative Knee Society (KSS) function score was significantly lower in the metal allergy cohort (*p* < 0.001). Furthermore, the KSS Symptoms, Satisfaction, and Expectations subdomains were all lower in the metal allergy cohort, suggesting that patient notification of a metal allergy may be associated with less satisfaction after the ATR. That group also had decreased SF-12 mental component scores compared with the conventional group, as we found in our participants; however, in the study by Nam, there was significant improvement in the postoperative mental component scores of the hypoallergenic TKA group compared with the group with no metal allergies (*p* < 0.01). This suggests that being notified of a metal allergy may be associated with lower satisfaction after TKA. These data mean that despite the effectiveness of TKA, there is a subset of patients with metal allergies who express dissatisfaction after undergoing this elective procedure.

In this regard, and given the high number of hypoallergenic implants used in this study, it seems clear that the performance of the patch test is not justified in TKA patients who self-report metal allergies if they do not have clear allergy symptoms (either local effects, such as localized dermatitis, rash, erythema, and local pruritus, or systemic effects, such as generalized pruritus, asthma, or systemic dermatitis).

It is clear that psychiatric pathology negatively influences clinical outcomes after TKA [9,18,19,20,31,32,33]. Examining pre-ATR psychological distress is important because of the predictive utility of psychological variables in surgical outcomes [20]. Previous research has identified that health-related quality of life, including quality-of-life-related mental health, is affected in patients awaiting intervention by ATR [34]. Our data corroborate this. The patients in both groups who presented psychological distress had worse results on the questionnaires, but when the patients with and without distress in the hypoallergenic implant group were compared, the difference was statistically significant.

In the hypoallergenic TKA group, there were more patients with severe psychological distress. The reason for this issue is not known, but it seems that it could be related to the functioning of the humoral system in the face of allergy. In addition, we were able to corroborate that patients in the hypoallergenic TKA group presented lower results on the different questionnaires when they suffered from psychological distress and had unfavorable postoperative scores on the SF-12 mental health questionnaire according to the ANCOVA and PSM, and these findings were statistically significant. Similarly, WOMAC scores were also lower among patients with allergies. These data agree with the results of Levenson et al. [35]. Therefore, the worse clinical results of patients who received hypoallergenic implants seem to be related not to the implant itself but to the patients’ humoral state in response to the allergy and the psychological distress they experience. We believe that this aspect is fundamental in the pre-surgical evaluation of patients.

The likelihood of an increase in the demand for TKA in future years requires further investigation into the possible causes of TKA failure; thus, the uncertainty surrounding metal hypersensitivity and its potential effects on the results of arthroplasty require careful analysis. Currently, and in light of our results, we believe that interventions designed to improve the QoL (BMI (through specific diets by nutritionists), preoperative pain (by rehabilitation and/or pain unit)) and to reduce psychological suffering (psychological and/or psychiatric management) are indicated for patients planning to undergo TKA, particularly those with a positive patch test for metal allergy. In fact, we believe that such interventions should be included in the algorithm for patients with a positive patch test who will undergo a TKA.

When interpreting the findings of this study, we must take into account several limitations. First, it is a retrospective review of medical records data collected prospectively from a specific population in a particular geographic area and cannot perfectly represent the country in general. Second, different surgeons performed the procedures. Furthermore, patient satisfaction is intrinsically subjective and, undoubtedly, multifactorial in nature. Although the age, sex, and BMI of the patient do not seem to be related to patient satisfaction with the outcome of the intervention, other authors have suggested many factors that may be related to postoperative results but were not included in the current analysis; such potentially related factors include preoperative patient expectations, socioeconomic status, race, preoperative diagnosis according to the degree of osteoarthritis on the Ahlbäck or Kellgren–Laurence scale, number and type of previous surgeries, and other comorbidities. In future studies it is necessary to delve into the immunological aspects. Finally, the time elapsed since surgery was different in each patient, but in all cases, more than 14 months had elapsed between the intervention and the completion of the questionnaires; we believe that this duration is long enough to allow the influence of the type of prosthesis on QoL to stabilize and therefore not affect the conclusions. To develop a more complete understanding of this topic, our future research should include a greater number of these underlying factors related to patient satisfaction.

## 5. Conclusions

Patients who receive hypoallergenic TKA have lower scores on the QoL and functional capacity questionnaires (WOMAC, SF-12, and Euro-QoL-5D L-VAS) and worse psychological distress compared with those who receive conventional Cr–Co implants. Patients with hypersensitivity to metal in our study present greater psychological distress, a fact that worsens quality-of-life parameters. We believe that it is necessary to evaluate the psychological aspect in patients with knee osteoarthritis and metal allergies who are going to undergo TKA.

## Figures and Tables

**Table 1:** Psychological distress score.

Psychological Distress
**Psychiatric history**
0: no diagnosis of psychiatric pathology
1: diagnosis by family doctor
2: diagnosis by the Psychiatry Service
**Type psychiatric pathology**
0: no pathology
1: minor depression or dysthymia
2: major depression or psychotic disorder
**N^o^. and type of drugs administered to the patient**
0: no medication
1: take a minor drug
2: take a major drug
3: take two or more drugs
**Final score**
1: 3 points, mild psychiatric pathology
2: 4–5 points, moderate psychiatric pathology
3: 6–7 points, severe psychiatric pathology

**Table 2:** Descriptive characteristics of the study sample. The results are presented as the mean ± standard deviation (SD) or total value and percentage. Body Mass Index (BMI), Western Ontario McMaster Universities Osteoarthritis Index (WOMAC), Short Form 12 questionnaire (SF-12 questionnaire), The EQ-5D-5L questionnaire essentially consists of 2 pages: the EQ-5D descriptive system (Euro-Qol-5D) and Visual Analog Scale (VAS).

	Total *n* = 245	Conventional TKA *n* = 169	Hypoallergenic TKA *n* = 76	*p*-Value
Mean age (years)	73.66 ± 6.59	74.27 ± 6.75	72.30 ± 6.03	0.030
Women, n (%)	170 (69.40)	100 (59.2)	70 (92.1)	<0.001
BMI (kg/m^2^)	32.17 ± 4.82	32.20 ± 4.71	32.12 ± 5.08	0.912
Weight, n (%)				
Normo-weight (BMI 18.5–24.9)	10 (4.1)	7 (4.1)	3 (3.9)	0.943
Overweight (BMI 25–29.9)	72 (29.4)	48 (28.4)	24 (31.6)	0.614
Obesity (BMI >30)	163 (66.5)	114 (67.5)	49 (64.5)	0.647
Time after intervention (months)	32.31±11.207	34.48 ±11.56	27.49 ±8.666	<0.001
Psychiatric history, n (%)	50 (20.40)	29 (17.2)	21 (27.6)	0.060
Associated complications, n (%)	31 (12.7)	19 (11.2)	12 (15.8)	0.322
WOMAC				
Total	24.59 ± 19.79	22.89 ± 19.21	28.37 ± 20.66	0.045
Pain	4.23 ± 4.88	3.75 ± 4.63	5.27 ± 5.29	0.026
Rigidity	1.17 ± 1.92	1.07 ± 1.83	1.41 ± 2.10	0.212
Difficulty	20.58 ± 14.75	19.76 ± 14.79	22.34 ± 14.62	0.215
SF-12				
Physical	29.76 ± 9.93	30.62 ± 9.74	27.28 ± 10.58	<0.001
Mental	46.06 ± 9.76	47.79 ± 9.51	44.86 ± 11.37	<0.001
Euro-QoL-5D				
Index	0.71 ± 0.31	0.74 ± 0.30	0.65 ± 0.32	0.035
VAS	60.35 ± 32.83	63.27 ± 31.51	53.86 ± 34.94	0.038

**Table 3:** Normality test Kolmogorov–Smirnov.

Normality Test			
Kolmogorov-Smirnov	Statistical t	gl	Sig.
Implant	0.438	245	0.000

**Table 4:** Results of the ANCOVA statistical analysis for the two study groups (conventional and Euro-Qol-5D) after pairing the patients by age, sex, weight, previous psychiatric history and associated complications. The results are presented as the mean ± standard deviation (SD).

	Conventional TKA *n* = 169	Hypoallergenic TKA *n* = 76	*p*-Value
WOMAC			
Total	22.61 ± 18.68	29.00 ± 19.45	0.022
Pain	3.76 ± 4.81	5.24 ± 5.00	0.042
Rigidity	1.09 ± 1.89	1.36 ± 1.96	0.336
Difficulty	19.57 ± 13.52	22.75 ± 14.07	0.121
SF-12			
Physical	30.74 ± 9.37	27.05 ± 10.55	0.561
Mental	46.49 ± 9.25	44.32 ± 10.78	0.038
Euro-QoL-5D			
Index	0.74 ± 0.29	0.65 ± 0.30	0.041
VAS	62.57 ± 32.38	55.41 ± 33.73	0.138

**Table 5:** Values of the propensity score matching (PSM) statistical analysis for the two study groups (conventional TKA versus hypoallergenic TKA) and the questionnaires (WOMAC, SF-12, and EuroQol-5D) after pairing the patients by age, sex, weight, previous psychiatric history and associated complications. The results are presented as the mean ± standard deviation (SD).

	Conventional TKA *n* = 169	Hypoallergenic TKA *n* = 76	*p*-Value
WOMAC			
Total	19.08 ± 8.83	28.37 ± 20.66	0.003
Pain	2.93 ± 1.28	5.27 ± 5.29	0.002
Rigidity	1.13 ± 1.78	1.41 ± 2.10	0.416
Difficulty	15.01 ± 8.27	22.34 ± 14.62	0.001
SF-12			
Physical	30.09 ± 9.09	28.25 ± 10.48	0.067
Mental	46.02 ± 9.67	44.05 ± 10.01	0.05
Euro-QoL-5D			
Index	0.83 ± 0.08	0.65 ± 0.32	<0.001
VAS	67.50 ± 6.22	53.86 ± 34.94	0.005

**Table 6:** Descriptive characteristics of the final score for psychological distress of the whole sample (conventional and hypoallergenic TKA).

	Patients (Knees)	% Patients (% Knees)
Patients		
No psychological distress	170 (184)	78,3 (78,6)
3 points, slight	18 (20)	8,3 (8,5)
4–5 points, moderate	11 (12)	5 (5,1)
6–7 points, severe	18 (18)	8,4 (7,8)
Total	217 (234)	100
Losses	11	4,5

**Table 7:** Descriptive characteristics of psychological distress according to conventional and hypoallergenic implant treatment. The results are presented as the total value and percentage.

Final Score for Psychological Distress Per Implant						
		No Psychological Distress	3 Points, Slight	4–5 Points, Moderate	6–7 Points, Severe	Total
CONVENTIONAL IMPLANT	No. of patients (knees)	116 (134)	12 (14)	9 (9)	6 (6)	143 (163)
	% patients inside the implant (knees)	81.1 (82.2)	8.4 (8.6)	6.2 (5.5)	4.4 (3.7)	100
ANTIALLERGIC IMPLANT	No. of patients (knees)	44 (51)	6 (6)	2 (3)	12 (12)	64 (72)
	% patients inside the implant (knees)	68.7 (70.8)	9.3 (8.3)	3.1 (4.2)	18.9 (16.7)	100
TOTAL	No. of patients (knees)	170 (184)	18 (20)	11 (12)	18 (18)	217 (234)
	% patients inside the implant (knees)	78.3 (78.6)	8.3 (8.5)	5 (5.1)	8.4 (7.8)	100

**Table 8:** Pearson’s chi-square test for psychological distress according to conventional and hypoallergenic implant treatment.

	Value	gl	Asymptotic Sig.
Pearson’s Chi-square	8.254	3	0.041
Likelihood ratio	7.676	3	0.053
No. cases included	234		

**Table 9:** Results of Student’s t for independent samples for psychological distress in patients treated with conventional and hypoallergenic implants with respect to the results of the questionnaires (WOMAC, SF-12, and Euro-Qol-5D).

		CONVENTIONAL			HYPOALLERGENIC		
	Psychological Distress	Mean	Standard Deviation	*p*-Value	Mean	Standard Deviation	*p*-Value
Total WOMAC	Yes	28.10	21.885	0.109	41.76	23.212	0.000
	No	21.81	18.512		23.25	17.229	
SF-12 physical	Yes	30.705	7.393	0.965	25.269	10.048	0.015
	No	30.592	10.628		32.804	10.058	
SF-12 Mental	Yes	46.042	9.962	0.319	39.568	12.665	0.017
	No	48.518	8.287		47.527	9.819	
EQ Index	Yes	0.76	0.30	0.321	0.84	0.25	0.019
	No	0.69	0.27		0.71	0.28	
EQ VAS	Yes	46.55	29.884	0.002	35.14	33.600	0.013
	No	66.74	30.814		61.00	33.004	

## Abbreviations

TKA	total knee arthroplasty
QoL	Quality of life
WOMAC	Western Ontario McMaster Universities Osteoarthritis Index
SF-12 questionnaire	Short Form 12 questionnaire
Euro-Qol-5D L-VAS	The EQ-5D-5L questionnaire essentially consists of two pages: the EQ-5D descriptive system and the EQ visual analogue scale (EQ VAS).
ANCOVA	analysis of covariance
PSM	propensity score matching
Cr-Co	chromium–cobalt
BMI	body mass index
PS	TKA posterior stabilized
PHQ-15 questionnaires	Patient Health Questionnaire Spanish version.
MPPF	María del Pilar Peña Fernández, author
KSS	Knee Society Clinical Rating System questionnaire
